# Hepatitis B virus vaccination status and associated factors among health care workers in Shashemene Zonal Town, Shashemene, Ethiopia: a cross sectional study

**DOI:** 10.1186/s13104-017-2582-0

**Published:** 2017-07-06

**Authors:** Tsega-Ab Abebaw, Zewdie Aderaw, Bereket Gebremichael

**Affiliations:** 1 Department of Occupational Health, Higher Education Relevance and Quality Agency, P. O. Box 13386, Addis Ababa, Ethiopia; 2 Department of Public Health, College of Medicine and Health Science, Debre Markos University, P. O. Box 259, Debre Markos, Ethiopia; 3 Department of Nursing, College of Medicine and Health Sciences, Mada Walabu University, Bale, P. O. Box 302, Goba, Ethiopia

## Abstract

**Background:**

Hepatitis B virus (HBV) remains a major global health problem. More than three-quarters of HBV infection occur in Asia, the Middle East and Africa. Healthcare workers (HCWs) are at risk of acquiring HBV, hepatitis C (HCV) and human immunodeficiency virus (HIV) infections via exposure to patients’ blood and bodily fluids. HBV infection is a recognized occupational hazard, and non-immune health professionals are at risk of acquiring the infection from their work. This study was intended to assess the level of HBV vaccination status and factors affecting the vaccination status of health care workers in Shashemene Zonal Town.

**Methods:**

Institution based cross-sectional study was conducted and a simple random sampling technique was used to select study subjects. A total of 423 HCWs were enrolled in the study. A structured and pre-tested questionnaire was used to collect the required information through a face to face interview. Finally, data were processed and analyzed using Epi info version 7 and SPSS version 21. Both bivariate and multivariable logistic regression analyses were used to assess the effect of the various factors on vaccination status of HCWs. p value ≤0.05 at 95% CI was considered statistically significant.

**Results:**

Overall, 53 (12.9%) respondents were found to be fully vaccinated. The multivariable logistic regression showed that, those respondents who are female, had ≥10 years of work experience and those working at governmental health care institutions were significantly associated with vaccination status (AOR = 3.84, 12.51, 2.45 respectively).

**Conclusion:**

Our study revealed that vaccination status of subjects was below the WHO’s estimation of vaccination rate among HCWs in developing countries and was very poor when compared with other countries. This is a serious public health problem and challenge for a country with high prevalence of hepatitis B infection.

## Background

Although there is a vaccine against hepatitis B virus (HBV) that provides 90–100% protection since the year 1982 [1], HBV infection is one of the major diseases causing, serious public health problems in the world [2]. According to the most recent World Health Organization (WHO) estimate, two billion people worldwide have serologic evidence of past or present HBV infection and 360 million are chronically infected and at risk for HBV-related liver disease [3]. These chronically infected persons are at higher risk of death from HBV-related liver cancer or cirrhosis by approximately 25%, and over 4 million new acute clinical cases occur annually. The virus is responsible for approximately 1.5 million deaths worldwide each year, two-thirds of which are attributable to primary hepatic carcinoma following HBV infection [4].

HBV is highly contagious, 50–100 times more infectious than Human Immunodeficiency Virus (HIV) [1] and is transmitted by percutaneous or mucosal exposure to infected blood or other bodily fluids. HBV transmission has been observed with numerous forms of human contact: perinatal/mother-to-child, nonsexual, sexual, needle-sharing and occupational/health-care-related. The highest concentrations of infectious HBV are found in blood and serum. However, other serum-derived body fluids, such as semen and saliva, are also infectious [3].

Throughout the world, millions of healthcare professionals work in health institutions. It is estimated that 600,000–800,000 cut and puncture injuries occur among these professionals per year. In hospitals it is estimated that approximately 30 injuries occur per 100 beds per year [5]. Accidental occupational injuries to health care workers (HCWs) continue to be a significant problem in the healthcare system. The worldwide incidence of percutaneous injury with a sharp object among the HCWs is estimated to be 3 million per year; where there is a chance of four injuries per HCW could occur annually. Exposure to unsafe blood as a result of the injury carries the risk of infections such as HIV, HBV, and HCV. An exposure to such unsafe bodily fluids in work places can add up to a considerable percent of infections occurring among HCWs [6].

HBV is the greatest threat of infection for HCWs worldwide. It is one of the most infectious pathogens that health care worker encounter on the job. The risk of contracting hepatitis B by health care personnel is four times greater than that of the general adult population, among those who do not work in healthcare institutions [5]. HCWs in developing countries are at serious risk of infection from blood-borne pathogens because of the high prevalence of such pathogens in many poorer regions of the world; especially in endemic areas like sub-Saharan Africa [7].

The health care work force comprises of about 59 million people worldwide who are exposed to a variety of health and safety hazards daily. The occupational health of this significant group has long been neglected both organizationally and by governments [8].

HBV infection is common due to a lapse in the sterilization technique of instruments or improper hospital waste management, as 10–20% of health care waste is regarded as hazardous, and may create a variety of health risks. Among the health care personnel, HBV is transmitted by skin prick with infected, contaminated needles and syringes or through accidental inoculation of minute quantities of blood during surgical and dental procedures. They are considered to be substantial risk for acquiring or transmitting the virus because of the occupational contact with blood, blood products and other body fluids [4]. Each day thousands of HCWs around the world suffer accidental occupational exposures during their role of caring for patients. A tremendously high (84.6%) level of life time exposure to unsafe body fluids was reported by the HCWs in Hawassa City, Southern Ethiopia [6].

The prevalence of some infections in HCWs depends on the disease prevalence in the general population, and some reports show a relatively higher sero-positivity for HBV among the HCWs in the health facilities. Studies report a high prevalence of needle stick injuries among health professionals in Ethiopia. Such occupational injuries which lead workers to dissatisfaction and psychological trauma may reduce motivation of the workers and in turn may affect the quality of health care [6].

Ethiopia has a high prevalence of liver disease. These cases account for 12% of the hospital admissions and 31% of the mortality in medical wards of Ethiopian Hospitals [9]. A study conducted in Ethiopia among health professionals revealed a 9.7% prevalence of hepatitis B surface antigen (HBsAg) [10]. According to a study conducted in Bahir Dar city administration, North West Ethiopia, hepatitis B vaccination rates among health care workers were low. From the total of 370 respondents, only 20 (5.4%) reported that they took three or more doses of hepatitis B vaccine. Health care workers’ knowledge about hepatitis B infection and the hepatitis B vaccine were also low. This is concerning, as all health care workers should be knowledgeable about this vaccine. In this study, only 62% of health care workers were knowledgeable about hepatitis B vaccine [11]. Although some previous studies determined HBV vaccination status of HCWs and non HCWs, no study has attempted to discover factors associated with their vaccination status. Therefore, the aim of this study is to assess HBV vaccination status of HCWs and determine factors associated with vaccination status of HCWs in Shashemene Zonal Town, Oromia Region, Ethiopia.

## Methods

### Study design

Institution based cross-sectional study was conducted to assess HBV vaccination status and associated factors among HCWs in Shashemene Zonal Town.

### Study area and period

The study was conducted in Shashemene Zonal Town, Oromia Region, South Ethiopia from March 16, 2015 to March 31, 2015. The town is located at 250 kms away from the capital city, Addis Ababa due south with a total area of 12,960 sq km and a total population of 153,162. The city administration is divided into eight sub-cities. According to the city administration, Health office report: the public and private organizations which are involved in health care delivery include 1 referral hospital having 171 HCWs, 1 district hospital having 94 HCWs and 3 governmental health centers having 42 HCWs which makes the total number of HCWs in governmental institutions 307 and 1 health center having 19 HCWs, 4 special clinics having 15 HCWs, 6 higher clinics having 55 HCWs, 16 middle clinics having 97 HCWs and 8 primary clinics having 28 HCWs which collectively give a total of 214 non-governmental institution HCWs. Therefore there are a total of 521 HCWs in Shashemene Zonal Town.

### Source and study population

The source population was all health care workers working in health care institutions found in Shashemene Zonal Town, during the data collection period and study population were those health care workers selected by using simple random sampling technique.

### Sample size

The study employed single population proportion sample size determination formula. Fifty percent vaccination prevalence was considered with 95% CI, and 5% marginal error (where n is desired sample size, Z is value of standard normal variable at 95% confidence interval and, p is proportion of vaccination status of HBV among HCWs and d is marginal error which is 5%) was considered to calculate the sample size. Considering a non-response rate of 10%, the final sample size was calculated to be 423.

### Sampling procedure

Samples were taken from all health care institutions in the town, and the samples were allocated proportionally based on the number of HCWs in the institutions by using a simple random sampling technique. Workers payroll was used as a sampling frame. To determine how many samples to take from a single institution, the total sample size needed (423) was multiplied by the total number of HCWs in the institution and divided by the total number of HCWs in the town (521).

### Inclusion and exclusion criteria

#### Inclusion criteria

Health care workers in health care institutions of Shashemene Zonal Town during the study period were the candidates of the study.

#### Exclusion criteria

Health care workers who were not in the study area during data collection period due to maternity, annual or sick leave and field work were excluded.

### Variables

#### Dependent variable

HBV vaccination.

#### Independent variables

Age, sex, religion, marital status, occupation category, length of employment, type of institution, knowledge about HBV, perceived price of vaccine and availability of vaccine.

### Operational definitions

#### Health care workers

Those health workers, who do have contact with syringes, needles, other sharp materials, blood and bodily fluids by the virtue of their duties (all types of nurses, health officers, medical doctors, dentists, laboratory technologists, anesthetists and janitors).

#### Knowledge about HBV

The questionnaire included a total of 21 knowledge questions regarding HBV. There were 10 questions about routes of transmission, 7 questions about natural history and diagnosis and 4 questions about prevention methods. The 21 questions included both correct and incorrect statements. The HCWs were asked to answer each question with ‘true’, ‘false’ or ‘I don’t know’. Each correct answer was given a score of ‘1’ while an incorrect answer and questions answered as ‘I don’t know’ was given a score of ‘0’. The scoring range of the questionnaire was 21 (largest) to 0 (smallest). According to the total score obtained, knowledge of HCWs was classified into ‘Knowledgeable’ and ‘Not knowledgeable’. A total score of 15 and above out of 21 was considered as knowledgeable.

#### Vaccination status

Since Immunocompetent adults and children who have vaccine-induced anti-HBs levels of ≥10 mIU/mL 1–2 months after having received a complete, ≥3-dose hepatitis B vaccine series are considered sero-protected and deemed vaccine responders [12], in our study, the respondents were grouped into two categories: ‘Fully vaccinated’ and ‘Not fully vaccinated’. ‘Full vaccination’ status was considered when HCWs have received three or more doses of hepatitis B vaccine. Respondents who were considered as not ‘fully vaccinated’ were those partially vaccinated (took one or two doses of hepatitis B vaccine) and not vaccinated (have not received any dose of vaccine) HCWs.

### Data collection procedures

A structured questionnaire was used to collect the required quantitative information through a face to face interview. The data was collected by nurses who were educated at the graduate level. Four data collectors and one supervisor were recruited to conduct the study. Those HCWs on the night or weekend shifts were addressed according to their working shifts. The investigators were responsible for coordination and supervision of the data collection process.

### Quality assurance

First the questionnaire was developed in English and then translated into Amharic and Affan Oromo, and back to English by different persons to check for consistency. Five percent of the questionnaires were pre tested in health care workers of Ras Desta Hospital. Based on the pretest, questions were revised, edited, and those questions found to be unclear or confusing were removed prior to the actual study. Knowledge questions were checked for content validity by experts. All the data collectors held graduate degrees, and they were given a half day of training. The investigators collected the completed questionnaires every day and checked each for inconsistencies and omissions. Any format with a defect was rejected from the study.

### Data management and data analysis

After being coded, the data was entered using EPI info version 7 statistical software and analyzed using SPSS version 21 statistical package. Data cleaning was performed to check for frequencies, accuracy, consistencies, missed values and variables. Any logical or consistency errors identified during data entry were corrected after revision of the original completed questionnaire. The cleaned and edited data was prepared for appropriate statistical analysis. The mean, standard deviation and the proportion of the variables were computed. Both bivariate and multivariable logistic regression analyses were used to measure how the outcome variable (HBV vaccination status) depends on the covariate variables (knowledge about HBV, perceived price of vaccine, availability of vaccine and socio demographic variables). Variables that showed association in the bivariate analyses were taken into the multivariable regression model to control for confounding. The results of the analysis were presented using tables, charts and graphs. 95% CI and p value of less than 0.05 were considered as indicators of statistical significance.

## Results

A total of 410 HCWs were enrolled in the study with a response rate of 96.9%.

### Socio demographic characteristics

The mean age of the participants was 29.42 years with a SD of 6.65. The age range of HCWs was 41 years (range 18–59 years). Most of the participants, 258 (62.9%) were in the age group of 18–29 years. Males accounted for 164 (40%) of the study subjects, resulting in an overall male to female ratio of 2:3. Out of the total, 172 (42%) were Orthodox and 224 (54.6%) were married. Majority of the respondents 183 (44.6%) were nurses. From the total respondents, 260 (63.4%) were from governmental and the rest were from non-governmental health care institutions. The duration of service year varied from a minimum of <1 year to 38 years (Table 1).

**Table 1:** Socio-demographic characteristic of the respondents

Variables	Category	Number (n)	Percent (%)
Sex	Male	164	40
	Female	246	60
Age group	18–29	258	62.9
	30–39	114	27.8
	40–59	38	9.3
Religion	Orthodox	172	42
	Muslim	108	26.3
	Protestant	111	27.1
	Catholic	16	3.9
	Wakefeta	3	0.7
Marital status	Married	224	54.6
	Single	173	42.2
	Divorced	9	2.2
	Widowed	4	1
Occupation	Nurse	183	44.6
	Cleaner	87	21.2
	Laboratory	49	12
	Health officer (HO)	33	8
	General practitioner (GP)	23	5.6
	Midwife	22	5.4
	Specialist doctor	6	1.5
	Doctor of dental medicine (DDM)	4	1
	Anesthetist	3	0.7
Work experience in years	0–4	215	52.4
	5–9	117	28.5
	≥10	78	19.1
Total		410	100

Shashemene, Ethiopia March 16, 2015–March 31, 2015 (N = 410)

### Vaccination status of HCWs

The overall fully vaccinated respondents were found to be 53 (12.9%) and those who had ever been vaccinated were 66 (16.1%) from the total 410 respondents. Among the 66 ever vaccinated respondents, 53 (80.3%) were fully vaccinated or took the vaccine three times and the remaining 5 (7.6%) and 8 (12.1%) took two and one time respectively or partially vaccinated. Only one (1.5%) had checked status of immunity after taking the vaccine.

### Reasons for not being vaccinated

**Fig. 1:**
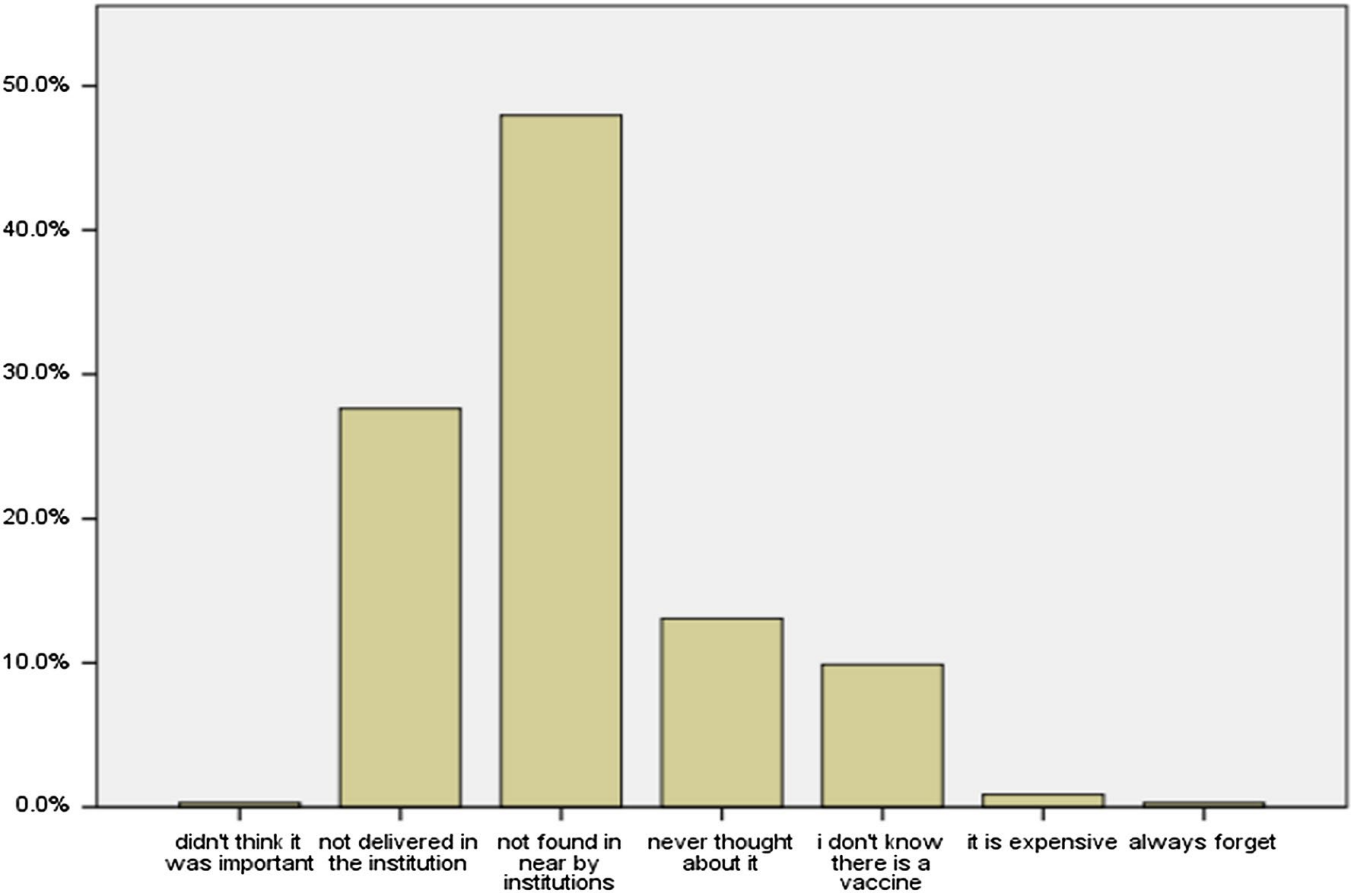
Reasons of the study participants for not being vaccinated for HBV Shashemene, Ethiopia March 16, 2015–March 31, 2015 (N = 344)

There were 344 unvaccinated respondents. The reasons for not being vaccinated varied from the highest 165 (48%), because the vaccine was not found in nearby institution, to the lowest 1 (0.3%), thinking it was not important, and 1 (0.3%) because of always forgetting to get the vaccine. The reasons for not being vaccinated are shown in Fig. 1.

None of the health care institutions have made available the vaccine and among the ever-vaccinated respondents, 10 (15.2%) had to pay to get the vaccine. The price of the vaccine was rated as expensive by 137 (33.4%), followed by 119 (29%) as very expensive and 36 (8.8%) as affordable, while 118 (28.8%) of respondents did not know the price. The majority of the respondents were found to be knowledgeable 257 (62.7%).

### Factors associated with vaccination status

Association between dependent variable and independent variables were tested with binary logistic regressions. The result showed that sex, age, marital status, work experience, type of institution and rated price, specifically between those who rated price of vaccine as affordable and those who didn’t know the price, had association with vaccination status of HCWs (Table 2).

**Table 2:** Factors associated with vaccination status of respondents

Variables	Categories	Fully vaccinated		COR (95% CI)	AOR (95% CI)
		Yes	No		
Sex	Male	14	150	1	1
	Female	39	207	*2* (*1.06, 3.85*)	*3.84* (*1.66, 8.92*)
Age	18–29	24	234	1	1
	30–39	19	95	*1.95* (*1.02, 3.73*)	0.54 (0.19, 1.56)
	40–59	10	28	*3.48* (*1.51, 8.03*)	1.09 (0.25, 4.70)
Religion	Orthodox	23	149	1	1
	Muslim	8	100	0.52 (0.22, 1.20)	0.67 (0.26, 1.70)
	Protestant	19	92	1.34 (0.69, 2.59)	1.34 (0.62, 2.92)
	Others	3	16	1.21 (0.33, 4.50)	2.40 (0.57, 10.11)
Marital status	Married	42	182	*3.67* (*1.83, 7.36*)	1.94 (0.86, 4.37)
	Others	11	175	1	1
Occupation	Nurse	28	155	1.46 (0.82, 2.60)	0.76 (0.37, 1.60)
	Others	25	202	1	
Experience	0–4	10	205	1	1
	5–9	21	96	*4.48* (*2.03, 9.89*)	*5.88* (*2.24, 15.42*)
	≥10	22	56	*8.05* (*3.60, 17.99*)	*12.5* (*3.10, 50.41*)
Knowledge	knowledgeable	38	219	1.60 (0.85, 3.01)	1.33 (0.61, 2.87)
	Not knowledgeable	15	138	1	1
Rated price	Affordable	8	28	1	1
	Expensive	22	115	0.67 (0.27, 1.66)	1 (0.35, 2.86)
	Very expensive	12	106	0.40 (0.14, 1.06)	0.41 (0.13, 1.25)
	Don’t know	11	108	*0.36* (*0.13, 0.97*)	0.34 (0.11, 1.07)
Type of institution	Governmental	43	217	*2.77* (*1.35, 5.70*)	*2.45* (*1.10, 5.46*)
	Non-governmental	10	140	1	1

Shashemene, Ethiopia March 16, 2015–March 31, 2015 (N = 410)

*NB* COR and AOR written in italics indicates statistical significance or p value <0.05

Then, multivariable logistic regression analysis was applied to control for confounders and identify the actual factors that are significantly associated with vaccination status of HCWs. After analysis, three variables were found to be significantly associated with vaccination status: sex, work experience and type of institution. Other socio demographic variables like age, occupation and marital status of respondents lost their association during the multivariable logistic regression model.

Female respondents were about four times as likely (AOR 3.84 [95% CI 1.66, 8.92]) (p = 0.002) to be fully vaccinated as compared to the male HCWs. Those who had a work experience 5–9 years were about six times more likely (AOR 5.88 [95% CI 2.24, 15.42]), and those who had ≥10 years were twelve times more likely (AOR 12.51 [95% CI 3.10, 50.41]) (p < 0.001) to be fully vaccinated than those who had a work experience of <5 years. The other variable that showed association was type of health institution. Those who work at governmental health care institutions are two times more likely (AOR 2.45 [95% CI 1.10, 5.46]) (p = 0.029) to be fully vaccinated than that of respondents from non-governmental health care institutions.

## Discussion

### Vaccination status

The World Health Organization has estimated that mean HBV vaccination rate among HCWs ranges from 18 to 39% in developing countries to 67–79% in developed countries [13]. However, the overall fully vaccinated respondents in our study was found to be 53 (12.9%) from a total of 410 respondents. Therefore, the percentage of fully vaccinated HCWs in Shashemene Zonal Town (12.9%) was found to be lower when compared to WHO’s estimation.

The result in this study was found to be higher when compared to other studies done in Ethiopia. A study conducted among HCWs of Bulle Hora Woreda, Southern Oromia, revealed that only 2 (1.2%) of the HCWs were vaccinated [14], and a study in St. Paul’s and Zewditu hospital, Addis Ababa showed 9 (3.5%) had history of vaccination even though none of them were fully vaccinated [15]. A study in Harar also showed only 15 (4.7%) of the respondents were fully vaccinated against HBV [16]. Another study conducted in Bahir Dar City Administration, North West Ethiopia showed low vaccination status among HCWs. According to the study 20 (5.4%) reported that they were fully vaccinated [11]. The disparity in vaccination rates in our study compared to the others conducted in Ethiopia could be due to the time they were conducted. With the exception of the study in Bahir Dar, all studies mentioned were conducted 3–5 years prior to our study. Another reason could be the vaccination campaign done by the Zonal Health Bureau among Shashemene Referral Hospital stuffs in the year 2012.

Comparison of our result with a study conducted in Georgia on health professionals showed a proportional result in which, 37 (12%) of the HCWs had been vaccinated against HBV [17]. Otherwise, it is very low when compared to 106 (53%) of HCWs at a tertiary care hospital in Pakistan who had received the vaccine [18] and a study in Iraq in which 45% of the respondents were vaccinated [4]. Another study from Iraq also showed relatively high percentage of HCWs vaccination. It showed 71 (65.7%) of the respondents had been vaccinated, from which 40 (37%) had completed taking the vaccine [19]. A study in India also showed 35% of the laboratory technicians were fully vaccinated [20]. Another study in tertiary teaching hospital in Nigeria revealed 162 (50.6%) of the respondents had ever received the vaccine while 116 (36.25%) had completed [8]. A study in a tertiary care hospital in India showing, 146 (57.5%) of the HCWs were vaccinated [13] and a study in Kuwait showing, 74.7% of the respondents have actually ever received the vaccine while 84.0% of them had completed [21]. The reasons for the lower rate of fully vaccinated HCWs in our study compared with other studies cannot be completely discerned. However, the difference in demographic characteristics of the study population, the difference in availability of the vaccine in these countries, and preliminary benefits due to the initiation of national programs of immunization in other countries might explain these discrepancies.

Only one (1.5%) had checked their status of immunity after taking the vaccine in our study. This is comparable with study in Iraq, in which no one of the respondents had checked for status of immunity after vaccination [19]. This might be due to the absence of a laboratory test in the town and health institutions nearby to detect antibodies against HBV.

### Reasons for not being vaccinated

Although a national hepatitis B vaccination policy is in place, and groups covered by this policy include infants and health care workers [22], is in place, remarkably majority (83.9%) of the respondents in our study have not been vaccinated. The reasons for not taking the vaccine by the respondents were, 165 (48%) because the vaccine was not found in nearby institution and 95 (27.6%) not delivered in the institution, which gives an overall 75.6% due to unavailability of the vaccine which is much higher than a study conducted in India showing 31.1% were not vaccinated due to same reason [13], 45 (13.1%) gave the reason ‘never thought about it’ which was lower than 21.7% of respondents of a study in Pakistan who gave same reason and the study also revealed, 24.8% of the respondents were not vaccinated because of no felt need [23] while in our study, only 1 (0.3%) of the respondents gave the reason ‘it was not important’. Studies conducted in India, Pakistan and Kuwait showed 13.3, 5.5 and 19.5% of the respondents respectively were not aware of the vaccine [13, 21, 23] while in our study 34 (9.9%) of the respondents were not aware of the vaccine, which was comparable with the study in India and about two times higher and lower than the studies in Pakistan and Kuwait respectively. Other reasons mentioned in our study were, 3 (0.9%) because it was expensive, and the remaining 1 (0.3%) always forget to get the vaccine which was much lower than a study conducted in India which showed 5.6% of respondents forgot to take vaccine [13].

### Factors associated with vaccination status

Female respondents were about four times as likely (AOR 3.84 [95% CI 1.66, 8.92]) to be fully vaccinated as compared to the male HCWs in this study. Similarly, a study conducted at a tertiary care hospital in India showed higher vaccination rate among female HCWs (χ^2^ = 6.41, df = 1, p < 0.02) [13]. The difference in vaccination status may be explained by greater risk behaviors in males than females, and women are more concerned to their health. In addition women have higher risk perception than men. The other possible explanation could be level of awareness since women were relatively more knowledgeable.

With increasing duration of work experience, there was a corresponding increase in full vaccination status. In our study, those who had a work experience 5–9 years were about six times more likely (AOR 5.88 [95% CI 2.24, 15.42]), and those who had ≥10 years were twelve times more likely (AOR 12.51 [95% CI 3.10, 50.41]) to be fully vaccinated than those who had a work experience of <5 years. A study in Nigeria also revealed the same pattern as our study, though the finding was not statistically significant (p = 0.323) [8]. This might be due to the fact that most of the HCWs were unvaccinated when entering health care services, and they become more likely to be vaccinated as they spend more time in the service because the health care institutions have no regular program of vaccination for the HCWs.

In general, this observation is worrisome because the most productive and economically viable age group of the populations were found in the groups with the least full vaccination status.

While none of the health care institutions found in the town have availed the vaccine, the type of institutions in our study that respondents come from have a significant association with vaccination status. Those who came from governmental health care institutions are two times more likely (AOR 2.45 [95% CI 1.10, 5.46) to be fully vaccinated than respondents from non-governmental health care institutions. This may be due to the fact that vaccination campaigns done in the town were only focused on the referral hospital stuff, and out of 241, majority 135 (56%) of the governmental institution respondents were from the referral hospital.

## Strength and limitation

This study has several strengths. It tried to identify the factors associated with HBV vaccine status of HCWs in addition to determining their vaccination status, which had not been previously investigated by other researchers in Ethiopia. We have also adopted and modified a questionnaire that was used by previous studies. Our study also has limitations. For instance, HBV vaccination status was assessed based on self-report rather than anti-HBs status, which could bring certain bias to the study outcome.

## Conclusions

Our study revealed that vaccination rates of the study subjects was below the WHO’s estimation of vaccination rates among HCWs in developing countries and was very poor when compared with other countries. This is a serious public health challenge for a country with high prevalence of hepatitis B infection.

The reason for not getting vaccinated by three-fourths of the respondents was because the vaccine was either not delivered in the institution, or it was not found in nearby institution. Some gave the reason ‘never thought about it’ and some were not aware of the vaccine. These findings should also not be neglected.

Even if there is the same risk of acquiring HBV infection, respondents from non-governmental institutions had lower odds of being fully vaccinated than respondents from the governmental institutions. There was also a corresponding increase in the proportion of respondents that received the vaccine as per increasing duration of work experience, and women had a relatively higher chance of being vaccinated.

Despite the national hepatitis B vaccination policy in place which is supposed to cover vaccine availability for infants and HCWs, a substantial amount of HCWs in our study had to pay to get the vaccine.

## Recommendations

- HBV-vaccination programs for HCWs are needed and the Zonal Health Bureau should consider this.
- The Regional Health Bureau should offer the vaccine to HCWs free of charge by coordinating efforts from other concerned bodies.
- The Zonal Health Bureau should make sure that the vaccine is available at health care institutions of the zonal town.
- It is better if HCWs get vaccinated at the time of employment before they spend more risky years at the health care institutions. Therefore, the employing institutions should take the responsibility to do that.
- The Zonal health bureau should consider including the non-governmental HCWs during the development or implementation of vaccination programs.
- A further study with better design and laboratory confirmation of vaccination status should be done in a large group of HCWs.

## Abbreviations

DDM: doctor of dental medicine
GP: general practitioner
HBV: hepatitis B virus
HBsAg: hepatitis B virus surface antigen
HCV: hepatitis C virus
HCW: health care worker
HIV: human immunodeficiency virus
HO: health officer
WHO: World Health Organization
